# High Remnant Cholesterol Level Potentiates the Development of Hypertension

**DOI:** 10.3389/fendo.2022.830347

**Published:** 2022-02-09

**Authors:** Ming-Ming Chen, Xuewei Huang, Chengsheng Xu, Xiao-Hui Song, Ye-Mao Liu, Dongai Yao, Huiming Lu, Gang Wang, Gui-Lan Zhang, Ze Chen, Tao Sun, Chengzhang Yang, Fang Lei, Juan-Juan Qin, Yan-Xiao Ji, Peng Zhang, Xiao-Jing Zhang, Lihua Zhu, Jingjing Cai, Feng Wan, Zhi-Gang She, Hongliang Li

**Affiliations:** ^1^ Department of Cardiology, Renmin Hospital of Wuhan University, Wuhan, China; ^2^ Department of Cardiology, Huanggang Central Hospital, Huanggang, China; ^3^ Institute of Model Animal, Wuhan University, Wuhan, China; ^4^ Physical Examination Center, Zhongnan Hospital of Wuhan University, Wuhan, China; ^5^ General Medical Department, China Resource and WireCo Wire Rope Co (CR & WISCO) General Hospital, Wuhan, China; ^6^ Basic Medical Laboratory, General Hospital of Central Theater Command, Wuhan, China; ^7^ Physical Examination Center, Xiaogan Central Hospital, Xiaogan, China; ^8^ Department of Cardiology, Zhongnan Hospital of Wuhan University, Wuhan, China; ^9^ Medical Science Research Center, Zhongnan Hospital of Wuhan University, Wuhan, China; ^10^ School of Basic Medical Science, Wuhan University, Wuhan, China; ^11^ Department of Cardiology, The Third Xiangya Hospital, Central South University, Changsha, China; ^12^ Department of Neurology, Huanggang Central Hospital, Huanggang, China; ^13^ Huanggang Institute of Translational Medicine, Huanggang, China

**Keywords:** hypertension, systolic blood pressure, diastolic blood pressure, retrospective cohort, remnant cholesterol

## Abstract

**Background:**

Emerging evidence suggests an association between remnant cholesterol (RC) and vascular damage and hypertension. However, this association has not been explored in a large-scale population in China, and a temporal relationship between RC and hypertension also needs to be investigated.

**Methods:**

We conducted a retrospective cross-sectional study in 2,199,366 individuals and a longitudinal study in 24,252 individuals with repeated measurements of lipid profile and blood pressure in at least a 3-year follow-up. The logistic model was used to explore the association between lipid components and hypertension in the cross-sectional analysis. The Cox model was used to analyze the association between high RC (HRC) at baseline and the subsequent incidence of hypertension or the association between hypertension at baseline and incidence of HRC. The cross-lagged panel model was applied to analyze the temporal relationship between RC and hypertension.

**Results:**

RC level as a continuous variable had the highest correlation with hypertension among lipid profiles, including RC, low-density lipoprotein cholesterol, total cholesterol, non-high-density lipoprotein cholesterol, and triglycerides, with an odds ratio of 1.59 (95% confidence interval: 1.58–1.59). In the longitudinal cohort, HRC at baseline was associated with incident hypertension. We further explored the temporal relationship between RC and hypertension using the cross-lagged analysis, and the results showed that RC increase preceded the development of hypertension, rather than vice versa.

**Conclusions:**

RC had an unexpected high correlation with the prevalence and incidence of hypertension. Moreover, RC increase might precede the development of hypertension, suggesting the potential role of RC in the development of hypertension.

## Introduction

As a hallmark of metabolic disorders, dyslipidemia often occurs together as a cluster of interconnected metabolic components, including obesity, hypertension, and diabetes (1, 2). It is well established that the coexistence of dyslipidemia and hypertension accelerates vascular damage and facilitates the development of cardiovascular diseases (CVD) (3). However, which component may lead to the other metabolic derangement progression is not well characterized. Epidemiological studies have revealed that traditional lipid profiles [e.g., low-density lipoprotein cholesterol (LDL-C), triglyceride (TG), total cholesterol (TC)] were associated with arterial stiffness measured by pulse-wave velocity in patients with hypertension, which is a marker of subclinical vascular damage (4–6). A recent cross-lagged path analysis suggested that increased arterial stiffness might precede increased blood pressure, indicating that vascular damage induces hypertension, rather than vice versa (7). Mechanically, dyslipidemia may cause vascular damage by impaired endothelial nitric oxide synthase activation, vascular inflammation, and increased oxidative stress (8–10). The joint evidence indicated that there may be a temporal relationship between dyslipidemia and hypertension. Identifying the relationship and controlling modifiable lipid levels have critical clinical implications regarding the management of hypertension.

Remnant cholesterol (RC) is a class of cholesterol content of triglyceride-rich lipoproteins composed of chylomicron remnants, very-low-density lipoprotein (VLDL), and intermediate-density lipoprotein (IDL) (11). Emerging observational evidence has shown that RC was associated with incident atherosclerotic diseases independent of traditional lipid profiles (11–13). RC had higher penetration into the arterial wall than traditional lipids and elicited arterial wall inflammation as well as low-grade systemic inflammation, further supporting the contribution of RC to vascular damage and atherosclerosis (14, 15). There were preliminary studies based on the small number of individuals that showed high RC (HRC) was associated with higher central systolic blood pressure and the incidence of hypertension (16–18). However, the abovementioned studies focused on a single-direction relationship between RC and hypertension, but the temporal relationship between these two entities has not been elucidated.

Therefore, we conducted a retrospective cross-sectional study in 2,199,366 individuals to explore the association between the RC levels and hypertension. Also, we performed a longitudinal study in 24,252 individuals with repeated RC levels and blood pressure in at least a 3-year follow-up. Using a cross-lagged analysis, we were able to explore the temporal relationship between RC levels and hypertension.

## Methods

### Study Population

**Figure 1:**
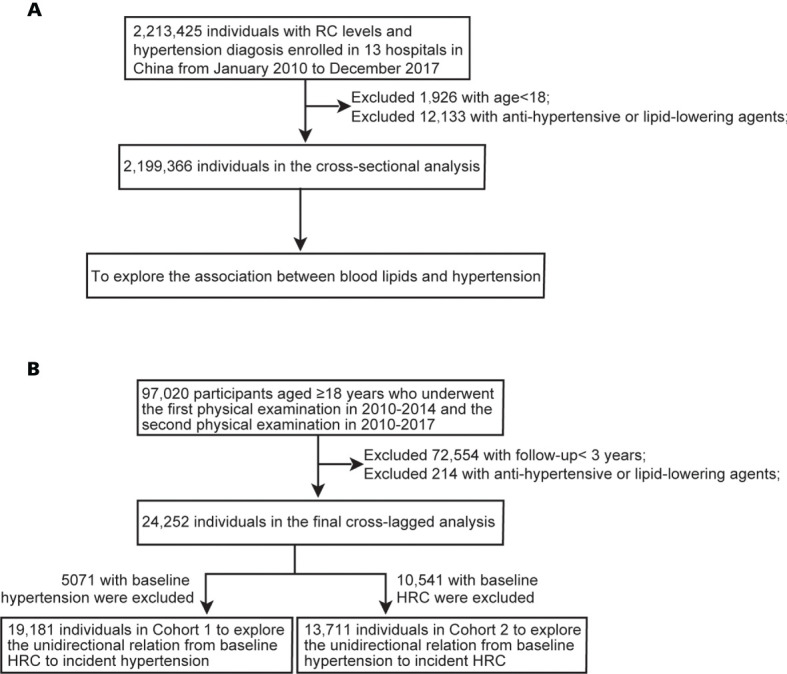
Flow chart of participants. **(A)** Flow chart of participants in the cross-sectional analysis. **(B)** Flow chart of participants in the longitudinal analysis. RC, remnant cholesterol; HRC, high remnant cholesterol.

This multicentered retrospective study includes 13 health management centers in China between January 2010 and December 2017. Participants in the health examinations were voluntary. Initially, 2,213,425 individuals with data of blood pressure and blood lipid, including TG, TC, LDL-C, and high-density lipoprotein cholesterol (HDL-C) were enrolled, and after excluding 1,926 individuals aged <18 years and 12,133 individuals with a previous history of antihypertensive or lipid-lowering agents, 2,199,366 individuals were involved in the cross-sectional analysis (**Figure 1A** **)**.

In the longitudinal cohort, 97,020 participants aged ≥18 years who underwent the first physical examination in 2010–2014 and the second physical examination in 2010–2017 were included (**Figure 1B**). 72,554 with follow-up <3 years and 214 with antihypertensive or lipid-lowering agents during follow-up were excluded. Finally, a total of 24,252 participants with repeated data were involved in the analysis of the bidirectional association between RC level and blood pressure. The outcome was determined according to the physical examination data from 2015 to 2017. To identify the association between baseline RC levels and incident hypertension during follow-up, we excluded participants with hypertension at baseline in Cohort 1. Similarly, we excluded participants with HRC at baseline in Cohort 2 to explore the association between baseline hypertension and incident HRC.

This study was approved by the central ethics board in Renmin Hospital of Wuhan University, followed by the acceptance with the ethics center in each collaborating hospital. Ethics committees granted a waiver of the requirement for documentation of informed consent for just analyzing existing data after anonymization without individual identification.

### Anthropometric and Laboratory Data

All participants had undergone comprehensive anthropometric measurements and clinical examinations by professional and experienced medical teams in each hospital. Height (cm) and body weight (kg) were measured to the nearest 0.1 kg and 0.1 cm, respectively, in subjects wearing light clothing without shoes. Waist circumference (cm) was measured to the nearest 0.1 cm at the midpoint between the lowest rib margin and iliac crest by a flexible anthropometric tape. Blood pressure (mmHg) was measured by mercury sphygmomanometers or electronic sphygmomanometers after individuals rested in a seated position for a minimum of 5 min (19). The measurement was carried out three times in succession at intervals of 1 min, and the average value of the measurement results was taken. Blood routine tests and biochemical tests were conducted in fasting state measured according to standard protocols and guidelines at a certified laboratory. TG was measured by the enzymatic colorimetric GPO-PAP method; TC was measured by the enzymatic-cholesterol oxidase peroxidase method; LDL-C and HDL-C were measured by direct homogenous assay (20). Body mass index (BMI) was calculated as weight divided by height square (kg/m^2^). Mean arterial blood pressure (MAP) was computed as 1/3 SBP+ 2/3 DBP. RC levels were calculated as TC minus HDL-C minus LDL-C (16). Non-high-density lipoprotein cholesterol (Non-HDL-C) was calculated as TC minus HDL-C (21). Past medical history of diseases and antihypertensive and lipid-lowering agents were obtained based on the self-reported history in the physical examination record.

### Diagnostic Criteria

According to 2018 Chinese hypertension management guidelines, hypertension was defined as SBP ≥ 140 mmHg, or DBP ≥ 90 mmHg, or a medical history of hypertension (22). Based on the joint consensus statement from the European Atherosclerosis Society and European Federation of Clinical Chemistry and Laboratory Medicine, HRC was defined as fasting RC ≥ 0.8 mmol/l and normal RC (NRC) is defined as fasting RC < 0.8 mmol/l (23). The diagnostic criteria for dyslipidemia were based on the guidelines for preventing and treating dyslipidemia in Chinese adults (24). Diabetes was defined as fasting blood glucose (FBG) ≥7.0 mmol/l, 2-h postprandial glucose ≥ 11.1 mmol/l, personal history of diabetes, or the use of hypoglycemic drugs (25, 26). Metabolic syndrome (MetS) was defined by the presence of at least three of the following components: (1) elevated waist circumference (≥90 cm for men and ≥80 cm for women); (2) elevated TG (≥1.70 mmol/l) or with drug treatment for elevated TG; (3) decreased HDL-C (<1.0 mmol/l for men and <1.3 mmol/l for women) or with lipid-lowering drug use for reduced HDL-C; (4) elevated blood pressure (≥130/85 mmHg) or with drug use for hypertension; and (5) elevated fasting glucose (≥5.6 mmol/l) or drug use for elevated glucose (27, 28). Fatty liver disease was defined by the evidence of hepatic steatosis on imageological examination (29, 30). Coronary heart disease and stroke were derived from the self-reported medical history.

### Statistical Analysis

Data were analyzed in R-3.6.3 (R Foundation for Statistical Computing, Vienna, Austria) and SPSS Statistics (version 23.0, IBM, Armonk, NY, USA). Continuous and categorical variables were presented as mean ± standard deviation (SD) and number with percent frequency, respectively. Comparisons between two groups were performed by Student’s t-test for normally distributed variables and the Wilcoxon rank-sum test for non-normally variables and the chi-square test or Fisher’s exact test for categorical variables. Statistical significance was considered as two-sided p < 0.05.

The association between the prevalence or the incidence of hypertension and continuous or categorical RC levels was analyzed using the logistic regression model in the cross-sectional population. Cox proportional hazard regression was conducted to explore the association between the categorical RC levels and incidence of hypertension or the association between hypertension and HRC occurrence in two cohorts. Age, sex, and heart rate were adjusted in Model 1. Age, sex, heart rate, BMI, FBG, leukocyte count (LEU), alanine aminotransferase (ALT), and serum creatinine (Scr) were adjusted in Model 2. Missing data with a proportion of missing of <50%, including heart rate, BMI, FBG, LEU, ALT, and Scr, were filled based on the missForest procedure in R (31, 32). A random forest model based on the rest of the variables in the dataset was constructed to predict the missing values with an estimation of the internally cross-validated errors (31). Odds ratio (OR) and hazard ratios (HR) with 95% confidence intervals (CI) were calculated. Statistical significance was considered as two-sided p < 0.05.

### Cross-Lagged Analysis

Cross-lagged path analysis, a statistical approach to explore the temporal relationship between variables over time, was performed to verify the bidirectional relationship between RC level and hypertension in our study (7). Variables measured repeatedly at two-time points were used in the models. According to the classification of the guideline, hypertension was classified into four grades, including none, grade 1, grade 2, and grade 3. None was defined by SBP < 140 mmHg, and DBP < 90 mmHg; grade 1 was defined by 140 mmHg ≤ SBP <160 mmHg, or 90 mmHg ≤ DBP <100 mmHg; grade 2 was defined by 160 mmHg ≤ SBP <180 mmHg, or 100 mmHg ≤DBP <110 mmHg; and grade 3 was defined by SBP ≥ 180 mmHg, or DBP ≥ 110 mmHg (22). Before the cross-lagged analysis, both baseline and follow-up RC and blood pressure values were controlled by residual regression analyses and then standardized by Z-transformation (mean = 0, SD = 1). Baseline and follow-up variables were adjusted *via* regression residual analyses before cross-lagged analysis. Model 1 was adjusted for age, sex, and heart rate; model 2 was adjusted for age, sex, heart rate, BMI, FBG, LEU, ALT, and Scr. The analysis measured the effect size of RC level in phase 1 on subsequent hypertension in phase 2 (β1), the effect size of hypertension in phase 1 on subsequent RC level in phase 2 (β2). The comparison between β1 and β2 was analyzed by t-test.

### Sensitivity Analyses

Four sensitivity analyses were conducted to assess the robustness of the bidirectional association between RC and blood pressure *via* the cross-lagged panel analysis. In test I, we estimated the relationship between RC and blood pressure, including SBP, DBP, and MAP in the cross-lagged model. In test II, we estimated the temporal relationship between RC and blood pressure, including hypertension, SBP, DBP, and MAP in the cross-lagged model after further adjusting for LDL-C, age, sex, heart rate, BMI, FBG, LEU, ALT, and Scr. In test III, we explored the temporal relationship of RC with hypertension, SBP, DBP, and MAP in 21,527 participants with BMI < 28 kg/m^2^. The adjusted confounders were age, sex, heart rate, FBG, LEU, ALT, and Scr. In test IV, we estimated the temporal relationship between RC and blood pressure, including hypertension, SBP, DBP, and MAP in the cross-lagged model after adjusting for age, sex, heart rate, BMI, FBG, LEU, ALT, Scr, waist circumference, coronary heart disease, stroke, and fatty liver disease.

## Results

### Clinical and Laboratory Characteristics in the Cross-Sectional Analysis

Overall, 2,199,366 cases were included with a mean age of 46.18 (SD, 13.45) and 58.88% of males. The mean SBP was 122.65 (SD, 18.00) mmHg, the mean DBP was 76.99 (SD, 11.94) mmHg, and the mean RC was 0.83 (SD, 0.59) mmol/l. 23.73% had hypertension, and 40.38% suffered dyslipidemia. A comparison of participants across categorical RC revealed that those individuals with HRC (with a clinically relevant cut point at 0.8 mmol/l) had generally higher levels of BMI, FBG, and blood pressure, as well as a higher percentage of hypertension. Detailed characteristics are provided in **Table 1**.

**Table 1:** Clinical characteristics in total and stratified by RC levels in a cross-sectional analysis.

	Total N = 2199366	NRC N = 1226732	HRC N = 972634	*p-value*
**Age (year, mean (SD))**	46.18 (13.45)	45.07 (13.87)	47.59 (12.76)	<0.001
**Gender, male (%)**	1,294,944 (58.88)	644,591 (52.55)	650,353 (66.87)	<0.001
**Heart rate (bpm, mean (SD))**	71.04 (10.32)	70.98 (9.82)	71.16 (11.15)	<0.001
**BMI (kg/m^2^, mean (SD))**	24.15 (4.67)	23.45 (4.08)	25.06 (5.19)	<0.001
**WC (cm, mean (SD))**	82.70 (10.35)	80.46 (10.34)	85.56 (9.64)	<0.001
**SBP (mmHg, mean (SD))**	122.65 (18.00)	120.37 (17.48)	125.53 (18.22)	<0.001
**DBP (mmHg, mean (SD))**	76.99 (11.94)	75.26 (11.51)	79.16 (12.12)	<0.001
**FBG (mmol/L, mean (SD))**	5.44 (1.27)	5.28 (1.03)	5.65 (1.50)	<0.001
**TC (mmol/L, mean (SD))**	4.88 (0.96)	4.53 (0.82)	5.32 (0.94)	<0.001
**TG (mmol/L, mean (SD))**	1.70 (1.53)	1.18 (0.58)	2.37 (2.02)	<0.001
**LDL-C (mmol/L, mean (SD))**	2.67 (0.74)	2.62 (0.76)	2.73 (0.71)	<0.001
**Non-HDL-C (mmol/L, mean (SD))**	3.50 (0.96)	3.08 (0.79)	4.04 (0.89)	<0.001
**RC (mmol/L, mean (SD))**	0.83 (0.59)	0.46 (0.20)	1.31 (0.58)	<0.001
**ALT (IU/L, mean (SD))**	26.63 (24.58)	23.65 (22.00)	30.42 (27.03)	<0.001
**AST (IU/L, mean (SD))**	24.94 (16.50)	23.49 (14.23)	26.63 (18.65)	<0.001
**γ-GGT (IU/L, mean (SD))**	36.18 (46.57)	29.09 (35.78)	43.29 (54.40)	<0.001
**Scr (μmol/L, mean (SD))**	71.33 (18.64)	69.11 (18.52)	74.12 (18.40)	<0.001
**SUA (μmol/L, mean (SD))**	329.36 (92.31)	308.96 (87.27)	352.44 (92.43)	<0.001
**BUN (mmol/L, mean (SD))**	4.95 (1.45)	4.83 (1.39)	5.09 (1.50)	<0.001
**RBC (×10^12^/L, mean (SD))**	4.76 (0.58)	4.69 (0.63)	4.84 (0.50)	<0.001
**LEU (×10^9^/L, mean (SD))**	6.22 (1.74)	6.07 (1.65)	6.40 (1.84)	<0.001
**HGB (g/L, mean (SD))**	143.58 (15.80)	141.39 (16.00)	146.37 (15.08)	<0.001
**PLT (×10^9^/L, mean (SD))**	205.52 (57.04)	208.15 (57.41)	202.20 (56.40)	<0.001
**Type 2 diabetes (%)**	145,264 (6.65)	60,578 (4.97)	84,686 (8.76)	<0.001
**Hypertension (%)**	522,015 (23.73)	241,769 (19.71)	280,246 (28.81)	<0.001
**Dyslipidemia (%)**	888,089 (40.38)	315,556 (25.72)	572,533 (58.86)	<0.001
**MetS (%)**	698,646 (33.44)	269,358 (22.90)	429,288 (47.01)	<0.001

RC, remnant cholesterol; NRC, normal remnant cholesterol; HRC, high remnant cholesterol; SD, standard deviation; BMI, body mass index; WC, waist circumference; SBP, systolic blood pressure; DBP, diastolic blood pressure; FBG, fasting blood glucose; TC, total cholesterol; TG, triglycerides; LDL-C, low-density lipoprotein cholesterol; Non-HDL-C, non-high-density lipoprotein cholesterol; ALT, alanine aminotransferase; AST, aspartate transaminase; γ-GGT, γ-glutamyltranspeptidase; Scr, serum creatinine; SUA, serum uric acid; BUN, blood urea nitrogen; RBC, red blood cell; LEU, leukocyte count; HGB, haemoglobin; PLT, platelet count; MetS, metabolic syndrome.

p-values were calculated by Student’s t-test for normally distributed variables and the Wilcoxon rank-sum test for non-normal distributed variables, as well as the chi-square test or Fisher’s exact test for categorical variables.

### Association Between RC Levels and Hypertension in the Cross-Sectional Populations

To identify which lipid component may have the highest association with hypertension, the logistic regression model was applied to analyze the associations between hypertension and continuous levels of lipids, including LDL-C, TG, TC, Non-HDL-C, and RC. The risk of hypertension was increased with increased lipid levels, particularly in patients stratified by RC (**Table 2**). The crude OR for hypertension by continuous RC was 1.59 (95% CI: 1.58–1.59) (**Table 2**). The relationship was still positive after adjusting for age, sex, heart rate, BMI, FBG, LEU, ALT, and Scr. Consistently, HRC was also associated with hypertension with the OR of 1.34 (95% CI: 1.34–1.35) after adjusting for the same confoundings (**Table 3**).

**Table 2:** Association between lipid levels and hypertension in the cross-sectional analysis.

Model	Variables (continuous, per 1 mmol/L)	OR (95% CI)	*p-value*
Crude	RC	1.59 (1.58,1.59)	<0.001
	LDL-C	1.42 (1.42,1.43)	<0.001
	TC	1.35 (1.35,1.36)	<0.001
	TG	1.26 (1.25,1.26)	<0.001
	Non-HDL-C	1.48 (1.47,1.48)	<0.001
Model 1^a^	RC	1.48 (1.47,1.48)	<0.001
	LDL-C	1.33 (1.33,1.34)	<0.001
	TC	1.30 (1.30,1.31)	<0.001
	TG	1.23 (1.23,1.23)	<0.001
	Non-HDL-C	1.39 (1.39,1.40)	<0.001
Model 2^b^	RC	1.30 (1.29,1.31)	<0.001
	LDL-C	1.27 (1.27,1.28)	<0.001
	TC	1.24 (1.24,1.25)	<0.001
	TG	1.16 (1.16,1.16)	<0.001
	Non-HDL-C	1.29 (1.29,1.30)	<0.001

OR, odds ratio; CI, confidence interval; RC, remnant cholesterol; LDL-C, low-density lipoprotein cholesterol; TC, total cholesterol; TG, triglycerides; Non-HDL-C, non-high-density lipoprotein cholesterol.

a In Model 1, the adjustment factors included age, sex, and heart rate.

b In Model 2, the adjustment factors included age, sex, heart rate, body mass index, fasting blood glucose, alanine aminotransferase, leukocyte count, and serum creatinine.

**Table 3:** HRC was associated with hypertension in cross-sectional analysis.

Model	NRC	HRC	
	OR(95% CI)	OR(95% CI)	*p-value*
Crude	ref	1.65 (1.64,1.66)	<0.001
Model 1^a^	ref	1.52 (1.51,1.53)	<0.001
Model 2^b^	ref	1.34 (1.34,1.35)	<0.001

NRC, normal remnant cholesterol; HRC, high remnant cholesterol; OR, odds ratio; CI, confidence interval.

a In Model 1, the adjustment factors included age, sex and heart rate.

b In Model 2, the adjustment factors included age, sex, heart rate, body mass index, fasting blood glucose, alanine aminotransferase, leukocyte count, and serum creatinine.

### Baseline Characteristics of the Longitudinal Analysis

Given the tight association between RC and hypertension in the cross-sectional analysis, we further conducted the longitudinal analysis to verify the temporal relationship between RC and hypertension. There was a total of 24,252 participants included with a mean age of 45.15 (SD, 11.27) years and 63.38% of men at the baseline. The detailed clinical characteristics are described in **Table S1**. The mean SBP was 121.37 (SD, 16.82) mmHg, the mean DBP was 77.51 (SD, 11.28) mmHg, and the mean RC was 0.81 (SD, 0.59) mmol/l (**Table S1**). Of note, 5,071 (20.91%) participants had hypertension and 10,541 (43.46%) had HRC at baseline.

### Association Analysis of Baseline HRC and the Occurrence of Hypertension in Cohort 1

A total of 19,181 (59.96% of male) participants initially without hypertension were included in Cohort 1 for exploring the association between baseline lipids and incidence of hypertension during the follow-up (**Table S2**). During a mean follow-up of 4.01 years (SD: 1.00), there were 2,804 (14.62%) new hypertension cases. The incident rate of hypertension was higher in the HRC group (45.59 per 1,000 person-years) than that in the NRC group (30.93 per 1,000 person-years). After adjusting for age, sex, heart rate, BMI, FBG, LEU, ALT, and Scr in the Cox proportional hazard regression model, the HR was 2.00 (95% CI: 1.85–2.16) for the individuals with HRC when compared to those with NRC (**Table 4**).

**Table 4:** Baseline HRC was associated with subsequent hypertension in Cohort 1.

	NRC	HRC	
Incident cases of hypertension, n (%)	1,487 (13.04)	1,317 (16.94)	–
Incident rate (per 1,000 person-years)	30.93	45.59	–
**Model**	**HR(95% CI)**	**HR(95% CI)**	***P-value***
Crude	ref	2.23 (2.07,2.41)	<0.001
Model 1^a^	ref	2.10 (1.94,2.26)	<0.001
Model 2^b^	ref	2.00 (1.85,2.16)	<0.001

NRC, normal remnant cholesterol; HRC, high remnant cholesterol; HR, hazard ratio; CI, confidence interval.

a In Model 1, the adjustment factors included age, sex, and heart rate.

b In Model 2, the adjustment factors included age, sex, heart rate, body mass index, fasting blood glucose, alanine aminotransferase, leukocyte count, and serum creatinine.

### Association Analysis of Baseline Hypertension and the Subsequent HRC in Cohort 2

Conversely, we explored the association between hypertension at baseline and the subsequent elevation in RC by the Cox proportional hazard regression model. 13,711 (57.01% of male) participants initially without HRC were included in Cohort 2 (**Table S3**). During a mean follow-up time of 4.21 years (SD: 1.06), there were 5,371 (39.17%) new HRC cases. The incident rate of HRC was higher in the hypertension group (106.22 per 1,000 person-years) than that in the non-hypertension group (90.30 per 1,000 person-years). Using individuals without hypertension as the reference in the Cox model, the HR (95% CI) for incidence of HRC was 1.25 (95% CI: 1.17–1.35) in individuals with hypertension, after adjusting for age, sex, heart rate, BMI, FBG, LEU, ALT, and Scr. These data indicated that baseline hypertension may also contribute to subsequent increases in RC levels (**Table S4**).

### Cross-Lagged Path Analysis for RC Levels and Hypertension

**Figure 2:**
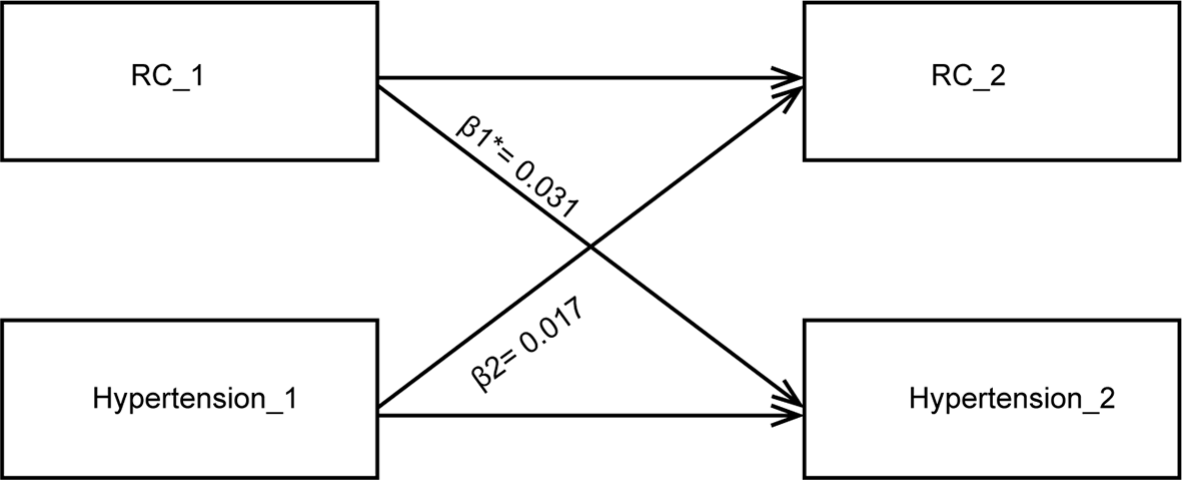
Cross-lagged path analysis for RC level and hypertension. The cross-lagged model is adjusted for age, sex, heart rate, body mass index, fasting blood glucose, alanine aminotransferase, leukocyte count, and serum creatinine. RC, remnant cholesterol; * indicates *P*<0.001 when comparing β1 vs. β2.

The positive associations between baseline RC and the subsequent incidence of hypertension and the baseline status of hypertension and RC level during the follow-up were established. The cross-lagged analysis was conducted in 24,252 participants with repeated measurement of RC and hypertension to further dissect the temporal relationship between these two entities. The mean follow-up period was 4.10 years (SD: 0.99). The standardized correlation coefficient of baseline RC and subsequent hypertension (β1 = 0.049; 95% CI: 0.037–0.061) was more significant than the standardized correlation coefficient of baseline hypertension and subsequent RC (β2 = 0.032; 95% CI: 0.021–0.044) with *p* < 0.001 for the difference in the crude model (**Table 5**). The effect sizes of two paths and the differences between two path coefficients remained significant in the two adjusted models (**Table 5** and **Figure 2**). The model fitting parameters of comparative fit indexes above all models were >0.900, indicating a relatively good fit to the observed data. These results indicated that the RC increase might precede the incidence of hypertension.

**Table 5:** Crude and adjusted cross-lagged standard regression coefficient of RC and blood pressure levels.

Model	β_RC-hypertension_(95% CI)	β_hypertension-RC_(95% CI)	*p-value* ^a^	CFI
Crude	0.049 (0.037,0.061)	0.032 (0.021,0.044)	<0.001	0.925
Model 1^b^	0.049 (0.037,0.061)	0.022 (0.010,0.034)	<0.001	0.930
Model 2^c^	0.031 (0.019,0.043)	0.017 (0.005,0.029)	<0.001	0.936

RC, remnant cholesterol; CI, confidence interval; CFI, comparative fit index.

a Test the difference of the cross-lagged path coefficients between β_RC-hypertension_ and β_hypertension-RC_ by the t-test.

b In Model 1, the adjustment factors included age, sex, and heart rate.

c In Model 2, the adjustment factors included age, sex, heart rate, body mass index, fasting blood glucose, alanine aminotransferase, leukocyte count, and serum creatinine.

### Sensitivity Analysis

In sensitivity analysis I, the standard regression coefficient of baseline RC to the follow-up SBP level (β = 0.035, 95% CI: 0.023–0.047) was significantly greater than the coefficient from the baseline SBP level to follow-up RC (β = 0.023, 95% CI: 0.011–0.035) with p < 0.001 for the difference after adjustment for age, sex, heart rate, BMI, FBG, LEU, ALT, and Scr, which implies that a high RC level at baseline might precede a subsequent increase in SBP during the follow-up, not vice versa. Correspondingly, the standard regression coefficient of baseline RC to the subsequent DBP level (β = 0.081, 95% CI: 0.069–0.093) was significantly greater than the coefficient from the baseline DBP level to subsequent RC (β = 0.024, 95% CI: 0.012–0.035) with p < 0.001 for the difference after adjustment for the same confounding factors. Similar patterns were shown between RC and MAP levels (**Table 6** and **Figure S1**). Also, consistent results were shown in sensitivity analyses II, III, and IV (**Table 6** and **Table S5**). These results consistently indicated that the RC increase may precede blood pressure increase, rather than vice versa.

**Table 6:** Cross-lagged standard regression coefficient of RC and blood pressure levels in the sensitivity analyses.

Sensitivity tests	Variables	Model	β_RC-blood pressure_(95% CI)	β_blood pressure-RC_(95% CI)	*p-value* ^a^	CFI
I	SBP	Model 1^b^	0.047 (0.035,0.059)	0.028 (0.016,0.040)	<0.001	0.920
		Model 2^c^	0.035 (0.023,0.047)	0.023 (0.011,0.035)	<0.001	0.928
	DBP	Model 1^b^	0.092 (0.081,0.104)	0.026 (0.014,0.037)	<0.001	0.922
		Model 2^c^	0.081 (0.069,0.093)	0.024 (0.012,0.035)	<0.001	0.930
	MAP	Model 1^b^	0.075 (0.064,0.087)	0.028 (0.017,0.040)	<0.001	0.916
		Model 2^c^	0.066 (0.054,0.077)	0.025 (0.013,0.037)	<0.001	0.925
II	Hypertension	Model 3^d^	0.030 (0.018,0.042)	0.020 (0.008,0.032)	<0.001	0.936
	SBP	Model 3^d^	0.034 (0.022,0.046)	0.025 (0.013,0.037)	<0.001	0.928
	DBP	Model 3^d^	0.077 (0.065,0.089)	0.019 (0.007,0.031)	<0.001	0.930
	MAP	Model 3^d^	0.063 (0.051,0.074)	0.023 (0.011,0.035)	<0.001	0.925
III	Hypertension	Model 4^e^	0.035 (0.022,0.048)	0.009 (-0.004,0.021)	<0.001	0.936
	SBP	Model 4^e^	0.039 (0.027,0.052)	0.016 (0.004,0.029)	<0.001	0.926
	DBP	Model 4^e^	0.088 (0.076,0.101)	0.018 (0.005,0.030)	<0.001	0.927
	MAP	Model 4^e^	0.071 (0.058,0.083)	0.018 (0.006,0.031)	<0.001	0.921

RC, remnant cholesterol; SBP, systolic blood pressure; DBP, diastolic blood pressure; MAP, mean arterial pressure; CI, confidence interval; CFI, comparative fit index.

a Test the difference of the cross-lagged path coefficients between β_RC-blood pressure_ and β_blood pressure-RC_ by the t-test.

b In Model 1, the adjustment factors included age, sex, and heart rate.

c In Model 2, the adjustment factors included age, sex, heart rate, body mass index, fasting blood glucose, alanine aminotransferase, leukocyte count, and serum creatinine.

d In Model 3, the adjustment factors included age, sex, heart rate, body mass index, fasting blood glucose, alanine aminotransferase, leukocyte count, serum creatinine, and low-density lipoprotein cholesterol.

e In Model 4, the adjustment factors included age, sex, heart rate, fasting blood glucose, alanine aminotransferase, leukocyte count, and serum creatinine.

## Discussion

To our best knowledge, this is the first study that revealed the association between RC level and hypertension based on a large-scale retrospective observational study of 2,199,366 population in China. The RC level has the highest correlation with hypertension among lipid profiles after adjusting for traditional confounders in the cross-sectional population. A positive relationship between HRC and the incident hypertension was also observed in the longitudinal cohort. The cross-lagged analysis indicated that RC increase might precede the development of hypertension, rather than vice versa.

Lipid metabolism and the development of hypertension are both complicated processes influenced by many different pathways (33–35). Since several metabolic disorders often occur together as a cluster of interconnected components characterized by obesity, hypertension, dyslipidemia, and insulin resistance, other metabolic risk factors may confound or mediate the association between RC and blood pressure. Thus, we adjusted common risk factors in our study, including metabolic factors BMI, waist circumference, and FBG. Consistent herewith, the positive association between RC and hypertension still existed in the logistic model, Cox model, and cross-lagged analysis, which further proved that elevated RC may precede the development of hypertension.

RC can be obtained by direct measurements or calculation through a standard lipid profile at the fasting or non-fasting state. Since fasting blood samples are routinely utilized to assess plasma lipid profiles, fasting RC has greater practical applicability than non-fasting RC. Different methods of measuring RC levels at the fasting state have been used to assess the association between RC and hypertension. In a prospective study in Japan, a positive association between RC detected directly by immunoseparation at baseline and incident hypertension in 681 subjects during a 10-year follow-up was observed (18). Due to the limited sample size and high rate of loss to follow-up, the conclusion was inconclusive. A recent cross-sectional study revealed that increased calculated RC level was significantly associated with higher central SBP in the Chinese population, but the temporal relationship between the two entities was not identified (16). Not only did our study reveal that baseline calculated RC at the fasting state was associated with the prevalence and incident hypertension, but also we used the cross-lagged model to untangle the unidirectional relationship from RC to hypertension in a large-size cohort in China. Consistently, the change of RC preceded the change of SBP, DBP, and MAP. In particular, RC increase also preceded the development of hypertension in participants with BMI < 28 kg/m^2^, indicating that RC-predisposed hypertension or increase in blood pressure may be independent of obesity. Although directly measured or calculated RC is associated with hypertension, no study has identified which one best predicts hypertension risk. Two meaningful investigations compared directly measured and calculated RC levels with the risk of ischemic heart disease and myocardial infarction, but the results were inconsistent that still cannot determine which one predicts the risk of CVDs better (36, 37). Further research is needed to compare the clinical value of different measurements of RC and reach a consensus on RC measurements.

Our study revealed that RC may be a potential culprit for the development of hypertension and CVD events. For many years, LDL-C has been recognized as a major risk factor for the incidence of CVDs, and reducing the level of LDL-C is the cornerstone in the treatment of atherosclerotic diseases, especially in patients with hypertension (38, 39). Yet clinical trials demonstrate persistent residual CVD risk despite aggressive LDL-C lowering. Focus on the management of RC level may be a plausible strategy for the residual risk reduction (40, 41). The evidence from our study showed that the RC level had a higher association with the prevalence of hypertension in all traditional lipid risk factors. In addition, an elevated RC level might precede the incident of hypertension after adjusting for traditional confounders. Prospective studies are warranted to explore the causal relationship between RC and the development of hypertension. Moreover, whether the reduction of the RC level halts or reverses the development of hypertension remains to be elucidated in future studies.

If we can prove that the early intervention of RC has a beneficial effect on blood pressure control and subsequent cardiovascular complications, identification of potential treatment strategies for controlling RC would be critical in the management of CVD. A *post hoc* analysis based on a recent study showed that Atorvastatin had a dose-dependent effect on RC control. This effect resulted in a significantly decreased risk in major adverse cardiovascular events independent of reducing LDL-C level (42). Pitavastatin and Pravastatin were also reported to reduce RC levels in patients with dyslipidemias (43). It is known that statins can lower blood pressure in patients with hypertension. Moreover, the potential mechanisms may be mediated by its beneficial effects on endothelial function and the renin–angiotensin system. Whether the impact on the blood pressure reduction is mediated by RC control remains to be further elucidated. Previous studies revealed that an increased RC level was positively associated with impaired flow-mediated endothelium-dependent dilatation (FMD) in the brachial artery. Application of Bezafibrate or Atorvastatin decreased the RC levels and markedly improved the FMD after a 4-week treatment (44). Impaired FMD reflects a reduction in nitric oxide production and endothelium dysfunction, which plays an essential role in the development of hypertension. The evidence mentioned above suggested that the lowering of RC may benefit the cardiovascular system, but the conclusion is preliminary. Besides lipid-lowing function, statin has multitarget effects that may intrigue pathways that regulate vascular tone, including the increase in bioavailability of nitric oxide, reduction in proinflammatory cytokines, and improvement in endothelial function (45, 46). Considering the vital role of RC in the development of CVD, treatment strategies targeting reducing RC warrant to be investigated.

The mechanistic basis of RC-associated hypertension is not fully understood, potentially involving vascular damage, inflammation, oxidative stress, the renin–angiotensin system, and insulin resistance. First, RC can penetrate arterial intima and promote increased secretion of various cytokines and adhesion molecules, leading to endothelial dysfunction, low-grade systematic, and vascular inflammation (33, 44, 47). Endothelial dysfunction and inflammation induce elevated systemic vascular resistance and impaired endothelium-dependent vasodilation because of an imbalance between vasoconstrictors and vasodilators and the subsequent development of hypertension. In addition, persistent inflammation may trigger the generation of reactive oxygen species and oxidative stress, which further impairs arterial function and has been associated with hypertension (48, 49). Second, studies also revealed that RC (mainly VLDL-C) stimulates aldosterone production, which increases sodium and water retention, potassium excretion, and blood volume, and thus trigging hypertension (50, 51). Third, elevated RC levels may be related to hyperinsulinemia and insulin resistance, which might stimulate sympathetic nervous system activity and the renin–angiotensin system, increase renal vascular resistance and renal sodium retention, and thus induce the development of hypertension (52–55). However, direct mechanisms that mediate RC-associated vascular injury need further investigation.

Several limitations should be noted in this study. First, our retrospective cohorts had inherent limitations in inferring the causal relationship between RC and hypertension; thus, the causal relationship warrants studying in prospective settings. Second, cross-lagged path analysis was able to verify the temporal relationship but was insufficient to verify the causality between RC and hypertension. Third, the mean follow-up period was 4–5 years, which was relatively short for the incidence of hypertension. Thus, this cohort is needed to be followed up in the long run. Fourth, limited information on smoking status, drinking, and insulin sensitivity may result in bias due to an insufficient adjustment of these confounders in the models. Fifth, the impact of the RC subtype, including chylomicron remnants, VLDL-C, and IDL-C, on the incidence of hypertension was not able to be evaluated due to the availability of data. Last, the study populations were based on national community-based health examinations but did not base on random sampling, and study data may underrepresent the rural populations of China.

## Conclusions

Our study showed that the RC level was associated with prevalent and incident hypertension. Importantly, elevated RC might precede the development of hypertension and the increase in blood pressure. These findings highlight the role of increased RC level in the development of hypertension and provide potential clinical relevance for controlling RC in hypertension management.

## Data Availability Statement

The original contributions presented in the study are included in the article/**Supplementary Material**. Further inquiries can be directed to the corresponding authors.

## Ethics Statement

This study was approved by the central ethics board of Renmin Hospital of Wuhan University and followed by the acceptance with the ethics center in each collaborating hospital. Ethics committees granted a waiver of the requirement for documentation of informed consent for just analyzing existing data after anonymization without individual identification.

## Author Contributions

In our research, M-MC, XH, and CX designed the study, analyzed the data, and wrote the first draft. X-HS, Y-ML, DY, HL, GW, G-LZ, ZC, and TS collected the data and contributed to the data analysis. CY, FL, and J-JQ wrote the codes for data analysis. Y-XJ, PZ, X-JZ, LZ, and JC revised the manuscript. FW, Z-GS, and HLi contributed equally, designed the project, edited the manuscript, and supervised the study. All authors contributed to the article and approved the submitted version.

## Funding

This work was supported by grants from the National Science Foundation of China (81970364, 81970070, 81970011, 81770053, 81870171, 82000299, 82170455, 82170595, 82170436), the Hubei Science and Technology Support Project (2019BFC582, 2018BEC473), the Medical Flight Plan of Wuhan University (TFJH2018006), the Henan Charity Federation Hepatobiliary Fund (GDXZ2021008), and the Funded Project of Youth Teacher (2042020kf0051).

## Conflict of Interest

The authors declare that the research was conducted in the absence of any commercial or financial relationships that could be construed as a potential conflict of interest.

## Publisher’s Note

All claims expressed in this article are solely those of the authors and do not necessarily represent those of their affiliated organizations, or those of the publisher, the editors and the reviewers. Any product that may be evaluated in this article, or claim that may be made by its manufacturer, is not guaranteed or endorsed by the publisher.
