# Supplementary material for: High Remnant Cholesterol Level Potentiates the Development of Hypertension
Source: Front Endocrinol (Lausanne). 2022 Feb 9;13:830347. doi: 10.3389/fendo.2022.830347 (PMC8863865; doi:10.3389/fendo.2022.830347)
Supplement: Supplementary file 1 [file DataSheet_1.pdf]

Supplemental Material

High Remnant Cholesterol Level Potentiates the Development of Hypertension

Table S1 Baseline characteristic of 24252 participants in longitudinal analysis

Table S2 Baseline characteristics of study variables in Cohort 1

Table S3 Baseline characteristics of study variables in Cohort 2

Table S4 Baseline hypertension was associated with subsequent HRC in Cohort 2

Table S5 Cross-lagged standard regression coefficient of RC and blood pressure levels in the IV sensitivity analysis

Figure S1 Cross-lagged standard regression coefficient of RC and blood pressure levels

Table S1 Baseline characteristic of 24252 participants in longitudinal analysis

	**Total (N=24252)**
**Age (year, mean (SD))**	45.15(11.27)
**Gender, male (%)**	15371(63.38)
**Heart rate (bpm, mean (SD))**	72.73(10.14)
**BMI (kg/m^2, mean (SD))**	24.12(3.28)
**WC (cm, mean (SD))**	83.67(10.31)
**SBP (mmHg, mean (SD))**	121.37(16.82)
**DBP (mmHg, mean (SD))**	77.51(11.28)
**FBG (mmol/L, mean (SD))**	5.50(1.19)
**TC (mmol/L, mean (SD))**	4.90(0.96)
**TG (mmol/L, mean (SD))**	1.78(1.59)
**LDL-C (mmol/L, mean (SD))**	2.71(0.73)
**RC (mmol/L, mean (SD))**	0.81(0.59)
**ALT (IU/L, mean (SD))**	27.80(24.12)
**AST (IU/L, mean (SD))**	26.99(14.90)
**γ-GGT (IU/L, mean (SD))**	36.07(41.01)
**Scr (μmol/L, mean (SD))**	71.50(15.98)
**SUA (μmol/L, mean (SD))**	344.11(90.75)
**BUN (mmol/L, mean (SD))**	5.15(1.31)
**RBC (×10^{12}/L, mean (SD))**	4.77(0.49)
**LEU (×10^9/L, mean (SD))**	6.02(2.00)
**HGB (g/L, mean (SD))**	144.49(14.83)
**PLT (×10^9/L, mean (SD))**	199.56(53.94)
**HRC (%)**	10541(43.46)
**Type 2 diabetes (%)**	1621(6.70)
**Hypertension (%)**	5071(20.91)
**Dyslipidemia (%)**	9975(41.13)
**MetS (%)**	8309(34.33)

Abbreviations: SD, standard deviation; BMI, body mass index; WC, waist circumference; SBP, systolic blood pressure; DBP, diastolic blood pressure; FBG, fasting blood glucose; TC, total cholesterol; TG, triglycerides; LDL-C, low-density lipoprotein cholesterol; RC, remnant cholesterol; ALT, alanine aminotransferase; AST, aspartate transaminase; γ-GGT, γ-glutamyltranspeptidase; Scr, serum creatinine; SUA, serum uric acid; BUN, blood urea nitrogen; RBC, red blood cell; LEU, leukocyte count; HGB, haemoglobin; PLT, platelet count; HRC, high remnant cholesterol; MetS, metabolic syndrome.

Table S2 Baseline characteristics of study variables in Cohort 1

	Total (N=19181)	NRC (N=11405)	HRC (N=7776)	*P-value* *
**Age (year, mean (SD))**	43.69(10.69)	43.56(10.81)	43.90(10.52)	0.032
**Gender, male (%)**	11500(59.96)	6164(54.05)	5336(68.62)	<0.001
**Heart rate (bpm, mean (SD))**	72.32(9.78)	73.22(8.94)	69.24(11.74)	<0.001
**BMI (kg/m^2, mean (SD))**	23.67(3.15)	23.18(3.11)	24.38(3.08)	<0.001
**WC (cm, mean (SD))**	82.24(10.06)	80.62(10.20)	84.30(9.48)	<0.001
**SBP (mmHg, mean (SD))**	115.18(11.04)	113.96(10.73)	116.98(11.24)	<0.001
**DBP (mmHg, mean (SD))**	73.60(8.05)	73.12(7.77)	74.31(8.39)	<0.001
**FBG (mmol/L, mean (SD))**	5.40(1.06)	5.30(0.90)	5.54(1.25)	<0.001
**TC (mmol/L, mean (SD))**	4.83(0.94)	4.51(0.81)	5.31(0.91)	<0.001
**TG (mmol/L, mean (SD))**	1.65(1.45)	1.20(0.57)	2.32(1.99)	<0.001
**LDL-C (mmol/L, mean (SD))**	2.68(0.72)	2.66(0.75)	2.71(0.67)	<0.001
**RC (mmol/L, mean (SD))**	0.77(0.56)	0.42(0.21)	1.28(0.53)	<0.001
**ALT (IU/L, mean (SD))**	26.37(21.36)	23.63(19.81)	30.40(22.86)	<0.001
**AST (IU/L, mean (SD))**	26.03(12.97)	23.94(10.54)	28.03(14.66)	<0.001
**γ-GGT (IU/L, mean (SD))**	32.94(36.90)	27.42(30.18)	39.97(42.99)	<0.001
**Scr (μmol/L, mean (SD))**	70.55(15.54)	68.51(15.32)	73.55(15.38)	<0.001
**SUA (μmol/L, mean (SD))**	335.46(88.69)	315.20(83.34)	354.86(89.33)	<0.001
**BUN (mmol/L, mean (SD))**	5.09(1.29)	5.01(1.28)	5.20(1.30)	<0.001
**RBC (×10^{12}/L, mean (SD))**	4.74(0.49)	4.66(0.48)	4.84(0.48)	<0.001
**LEU (×10^9/L, mean (SD))**	5.98(2.10)	5.85(1.51)	6.17(2.73)	<0.001
**HGB (g/L, mean (SD))**	143.42(14.89)	141.50(15.24)	146.32(13.85)	<0.001
**PLT (×10^9/L, mean (SD))**	200.74(53.92)	204.31(53.77)	195.45(53.71)	<0.001
**HRC (%)**	7776(40.54)	0(0.00)	7776(100.00)	<0.001
**Type 2 diabetes (%)**	966(5.05)	476(4.18)	490(6.33)	<0.001
**Hypertension (%)**	0(0.00)	0(0.00)	0(0.00)	-
**Dyslipidemia (%)**	7218(37.63)	3310(29.02)	3908(50.26)	<0.001
**MetS (%)**	4616(24.11)	1986(17.45)	2630(33.89)	<0.001

Abbreviations: NRC, normal remnant cholesterol; HRC, high remnant cholesterol; SD, standard deviation; BMI, body mass index; WC, waist circumference; SBP, systolic blood pressure; DBP, diastolic blood pressure; FBG, fasting blood glucose; TC, total cholesterol; TG, triglycerides; LDL-C, low-density lipoprotein cholesterol; RC, remnant cholesterol; ALT, alanine aminotransferase; AST, aspartate transaminase; γ-GGT, γ-glutamyltranspeptidase; Scr, serum creatinine; SUA, serum uric acid; BUN, blood urea nitrogen; RBC, red blood cell; LEU, leukocyte count; HGB, haemoglobin; PLT, platelet count; MetS, metabolic syndrome.

* *P-values* were calculated by student's t-test for normally distributed variables and the Wilcoxon rank-sum test for non-normal distributed variables, as well as the chi-square test or Fisher's exact test for categorical variables.

Table S3 Baseline characteristics of study variables in Cohort 2

	Total (N=13711)	Non-hypertension (N=11405)	Hypertension (N=2306)	*P-value* *
**Age (year, mean (SD))**	44.92(11.42)	43.56(10.81)	51.63(11.99)	<0.001
**Gender, male (%)**	7816(57.01)	6164(54.05)	1652(71.64)	<0.001
**Heart rate (bpm, mean (SD))**	73.45(9.14)	73.22(8.94)	74.56(10.01)	<0.001
**BMI (kg/m^2, mean (SD))**	23.55(3.22)	23.18(3.11)	25.35(3.18)	<0.001
**WC (cm, mean (SD))**	81.83(10.55)	80.62(10.20)	87.97(10.12)	<0.001
**SBP (mmHg, mean (SD))**	118.74(15.47)	113.96(10.73)	142.37(13.47)	<0.001
**DBP (mmHg, mean (SD))**	76.17(10.40)	73.12(7.77)	91.24(8.48)	<0.001
**FBG (mmol/L, mean (SD))**	5.37(0.99)	5.30(0.90)	5.71(1.31)	<0.001
**TC (mmol/L, mean (SD))**	4.54(0.82)	4.51(0.81)	4.71 (0.86)	<0.001
**TG (mmol/L, mean (SD))**	1.24(0.59)	1.20(0.57)	1.45(0.64)	<0.001
**LDL-C (mmol/L, mean (SD))**	2.70(0.77)	2.66(0.75)	2.90(0.80)	<0.001
**RC (mmol/L, mean (SD))**	0.42(0.21)	0.42(0.21)	0.44 (0.21)	<0.001
**ALT (IU/L, mean (SD))**	24.58(21.66)	23.63(19.81)	29.27 (28.66)	<0.001
**AST (IU/L, mean (SD))**	24.60(12.16)	23.94(10.54)	27.80(17.79)	<0.001
**γ-GGT (IU/L, mean (SD))**	29.23(32.17)	27.42(30.18)	37.87(39.20)	<0.001
**Scr (μmol/L, mean (SD))**	69.27(15.46)	68.51(15.32)	72.99(15.59)	<0.001
**SUA (μmol/L, mean (SD))**	321.90(85.06)	315.20(83.34)	354.75(85.78)	<0.001
**BUN (mmol/L, mean (SD))**	5.07(1.29)	5.01(1.28)	5.35(1.32)	<0.001
**RBC (×10^{12}/L, mean (SD))**	4.69(0.48)	4.66(0.48)	4.83(0.47)	<0.001
**LEU (×10^9/L, mean (SD))**	5.88(1.52)	5.85(1.51)	6.06(1.55)	<0.001
**HGB (g/L, mean (SD))**	142.46(15.24)	141.50(15.24)	147.22(14.33)	<0.001
**PLT (×10^9/L, mean (SD))**	203.62(53.89)	204.31(53.77)	200.18(54.39)	0.001
**HRC (%)**	0(0.00)	0(0.00)	0(0.00)	-
**Type 2 diabetes (%)**	708(5.17)	476(4.18)	232(10.07)	<0.001
**Hypertension (%)**	2306(16.82)	0(0.00)	2306(100.00)	<0.001
**Dyslipidemia (%)**	4277(31.19)	3310(29.02)	967(41.93)	<0.001
**MetS (%)**	3431(25.08)	1986(17.45)	1445(62.94)	<0.001

Abbreviations: SD, standard deviation; BMI, body mass index; WC, waist circumference; SBP, systolic blood pressure; DBP, diastolic blood pressure; FBG, fasting blood glucose; TC, total cholesterol; TG, triglycerides; LDL-C, low-density lipoprotein cholesterol; RC, remnant cholesterol; ALT, alanine aminotransferase; AST, aspartate transaminase; γ-GGT, γ-glutamyltranspeptidase; Scr, serum creatinine; SUA, serum uric acid; BUN, blood urea nitrogen; RBC, red blood cell; LEU, leukocyte count; HGB, haemoglobin; PLT, platelet count; HRC, high remnant cholesterol; MetS, metabolic syndrome.

* *P-values* were calculated by student's t-test for normally distributed variables and the Wilcoxon rank-sum test for non-normal distributed variables, as well as the chi-square test or Fisher's exact test for categorical variables.

Table S4 Baseline hypertension was associated with subsequent HRC in Cohort 2

	Non-hypertension	hypertension	
Incident cases of HRC, n (%)	4341(38.06)	1030(44.67)	
Incident rate (per 1000 person-years)	90.30	106.22	
**Model**	**HR(95% CI)**	**HR(95% CI)**	***P-value***
Crude	ref	1.20(1.12,1.29)	<0.001
Model 1*	ref	1.23(1.15,1.32)	<0.001
Model 2†	ref	1.25(1.17,1.35)	<0.001

Abbreviations: HRC, high remnant cholesterol; HR, hazard ratio; CI, confidence interval.

* In Model 1, the adjustment factors included age, sex and heart rate.

† In Model 2, the adjustment factors included age, sex, heart rate, body mass index, fasting blood glucose, alanine aminotransferase, leukocyte count, and serum creatinine.

Table S5 Cross-lagged standard regression coefficient of RC and blood pressure levels in the IV sensitivity analysis

**Variables**	**$\beta_{\text{RC-blood pressure}}$(95% CI)**	**$\beta_{\text{blood pressure-RC}}$(95% CI)**	***P-value*** *	**CFI**
Hypertension	0.025(0.013,0.037)	0.007(-0.005,0.019)	*<0.001*	0.944
SBP	0.031(0.019,0.043)	0.011(-0.001,0.023)	*<0.001*	0.933
DBP	0.068(0.056,0.079)	0.024(0.012,0.036)	*<0.001*	0.935
MAP	0.055(0.044,0.067)	0.020(0.008,0.032)	*<0.001*	0.930

Abbreviations: RC, remnant cholesterol; SBP, systolic blood pressure; DBP, diastolic blood pressure; MAP, mean arterial pressure; CI, confidence interval; CFI, comparative fit index.

* Test the difference of the cross-lagged path coefficients between $\beta_{\text{RC-blood pressure}}$ and $\beta_{\text{blood pressure-RC}}$ by the t-test.

The adjustment factors included age, sex, heart rate, body mass index, fasting blood glucose, alanine aminotransferase, leukocyte count, serum creatinine, waist circumference, coronary heart diseases, stroke, and fatty liver disease.

Figure S1

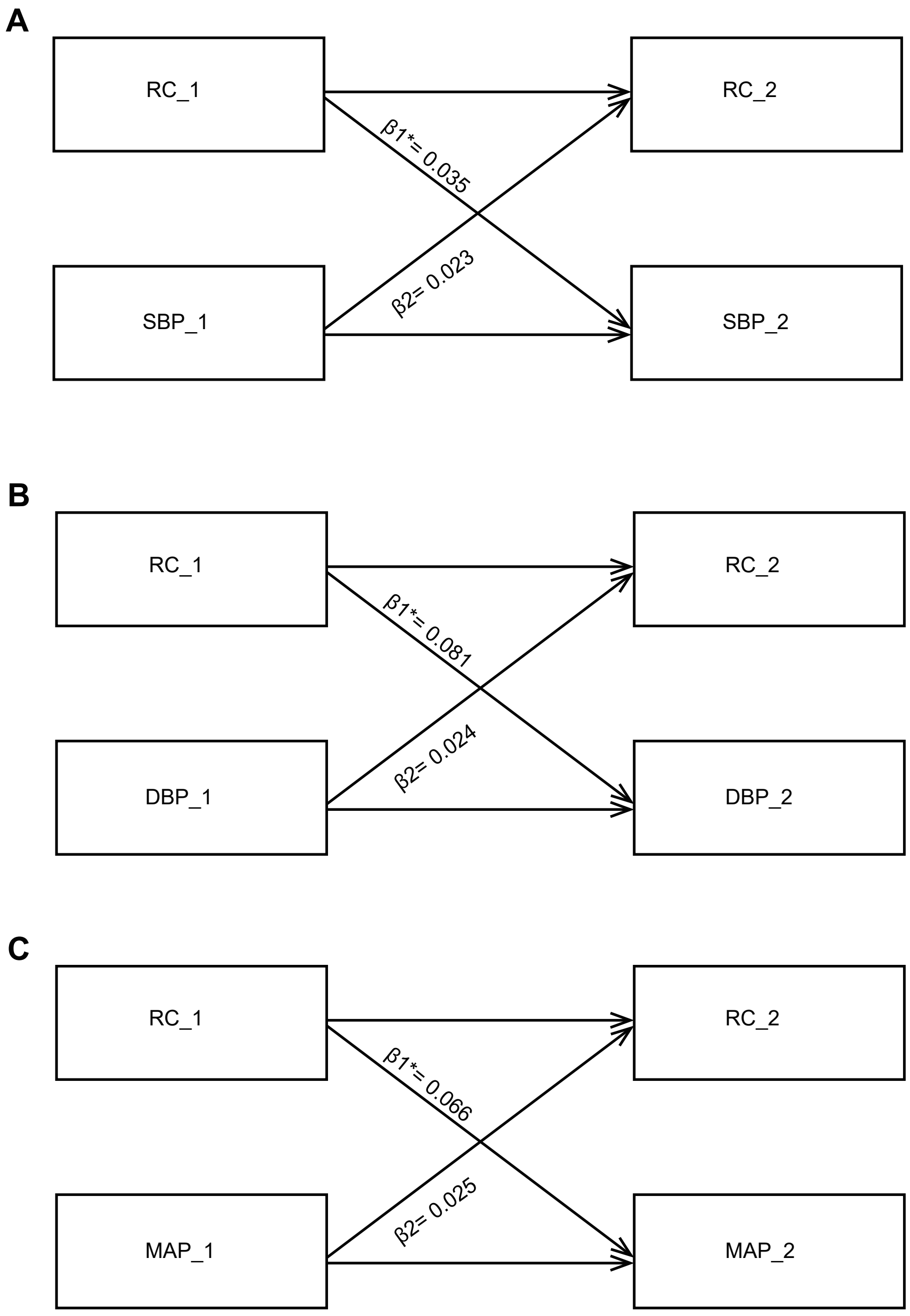

Figure S1 Cross-lagged standard regression coefficient of RC and blood pressure levels in the sensitivity analysis I. A,SBP; B, DBP; C, MAP The cross-lagged model was adjusted for age, sex, heart rate, body mass index, fasting blood glucose, alanine aminotransferase, leukocyte count, and serum creatinine. Abbreviations: RC, remnant cholesterol; SBP, systolic blood pressure; DBP,diastolic blood pressure; MAP, mean arterial pressure. * indicates *P*<0.001 when comparing β1 vs. β2.
